# The Rise of Fungi: A Report on the CIFAR Program *Fungal Kingdom: Threats & Opportunities* Inaugural Meeting

**DOI:** 10.1534/g3.120.401271

**Published:** 2020-04-17

**Authors:** Nicola T. Case, Joseph Heitman, Leah E. Cowen

**Affiliations:** * Department of Molecular Genetics, University of Toronto, Ontario, Canada, M5G 1M1 and; ^†^ Department of Molecular Genetics and Microbiology, Medicine, and Pharmacology and Cancer Biology, Duke University Medical Center, Durham, North Carolina, 27710

**Keywords:** fungal pathogens, antifungal resistance, medical mycology, plant-pathogenic fungi, wildlife pathogens

## Abstract

The first meeting of the CIFAR *Fungal Kingdom: Threats & Opportunities* research program saw the congregation of experts on fungal biology to address the most pressing threats fungi pose to global health, agriculture, and biodiversity. This report covers the research discussed during the meeting and the advancements made toward mitigating the devastating impact of fungi on plants, animals, and humans.

CIFAR is a Canadian-based global charitable organization dedicated to enabling research to tackle the most important questions facing science and humanity. The *Fungal Kingdom* program is one of four new research programs officially launched by CIFAR on July 1, 2019, as a result of a global call for ideas announced in November 2017. With an international team comprised of twelve fellows, five advisory board members, and co-directors Leah Cowen (University of Toronto, Canada) and Joseph Heitman (Duke University, USA), the *Fungal Kingdom* program is poised to develop new strategies to disarm the threats posed by fungi and harness their extraordinary potential.

Although often underappreciated, the contribution of fungi to life on Earth and human civilization is staggering. In the environment, fungi are preeminent degraders of organic matter, form mutually beneficial symbioses with 90% of plant species, enhance soil carbon sequestration, and prevent desertification (Řezáčová *et al.* 2017; Willis 2018). In human civilization, enzymes produced by fungi are crucial for fermentation, food manufacturing, bioremediation, and biofuel production (Strobel 2015; Willis 2018). Moreover, fungi produce secondary metabolites that are integral to modern medicine, such as antibiotics and immunosuppressive drugs that enable organ transplantation (Keller *et al.* 2005). While the beneficial role of fungi is clear, every kingdom has a dark side, and the devastating impact of fungi on human health, global food security, and biodiversity is growing (Fisher *et al.* 2012, 2016, 2018; Fones *et al.* 2017). Fungi infect billions of people worldwide and kill in excess of 1.5 million per year, a death toll on par with prominent bacterial and parasite pathogens, such as those causing tuberculosis and malaria (Brown *et al.* 2012a, 2012b). Alarmingly, the incidence of invasive fungal infections is increasing, and multidrug-resistant pathogens are spreading across the globe (Fisher *et al.* 2016, 2018). In tandem, fungi are causing epidemics in staple crops and extinctions in wild species, with mass mortalities of hibernating bats and amphibians (Fisher *et al.* 2016). Taken together, it is clear that fungi pose a devastating threat to our planet and society. Averting their catastrophic potential is contingent on understanding fungal biology and developing resistance-evasive strategies to protect humans, crops, and wildlife from infection. In an effort to address these threats, the CIFAR *Fungal Kingdom* program aims to tackle four grand challenges, which were the topic of the meeting: 1) understand forces driving the emergence, evolution, and spread of fungi impacting plants, animals, human health, and society; 2) identify mechanisms of fungal adaptation and interactions with hosts and other microbes; 3) understand the evolution of resistance to fungicides and antifungals across the fungal kingdom; and 4) develop novel strategies to thwart fungal disease.

## Understand forces driving the emergence, evolution, and spread of fungi impacting plants, animals, human health, and society

The fungi with the greatest capacity to become threats are those with high ‘evolutionary potential’ – the ability to rapidly adapt to new environments, overcome host defenses, or develop drug resistance (Taylor *et al.* 2017). Matthew Fisher (Imperial College London, England) proposed two guiding observations to understand the emergence, evolution, and spread of fungal pathogens. The first observation is that evolutionary hotspots for fungal pathogens of animals are marked by high pathogen genome diversity and infection-tolerant hosts. The second is that globalization drives fungal disease emergence, and as trade networks erode geographical barriers to pathogen transmission, we are generating a functional Pangaea. Fisher demonstrated these principles in his work tracing the origins and emergence of the human fungal pathogen *Cryptococcus gattii* and the amphibian fungal pathogen *Batrachochytrium dendrobatidis*. Fisher, in partnership with Christina Cuomo (Broad Institute, USA), discovered a new lineage of *C. gattii* (variety *gattii* five, VGV) in Zambia, Africa, in addition to four out of six of the world’s *C. gattii* species, implicating Sub-Saharan Africa as a center of *C. gattii* diversity (Farrer *et al.* 2019). Fisher inferred the spatiotemporal origins of the most devastating fungal panzootic to date, caused by *B. dendrobatidis*, to East Asia and dated the spread to the early 20^th^ century, coinciding with the global expansion of commercial trade in amphibians (O’Hanlon *et al.* 2018). He illustrated the impact of globalization on the emergence and spread of fungal pathogens and warned that pathogen re-contact will inevitably expand genetic diversity and affect virulence and antifungal resistance.

In addition to disseminating fungal disease, globalization can foster the emergence of fungal pathogens through hybrid speciation events as otherwise non co-existing fungi are brought into contact. Eva Stukenbrock (Kiel University, Germany) demonstrated a role for hybridization in the catalysis of new pathogenic *Zymoseptoria* species (Stukenbrock *et al.* 2012; Feurtey *et al.* 2019). Through sequencing of *Zymoseptoria pseudotritici* isolates from Iran, Stukenbrock observed highly unusual sequence diversity patterns, consistent with the emergence of *Z. pseudotritici* from a hybrid speciation event (Stukenbrock *et al.* 2012). Genomic analysis of other *Zymoseptoria* species, including the pervasive wheat pathogen *Zymoseptoria tritici*, revealed a similar diversity pattern indicating that hybridization occurs frequently between *Zymoseptoria* species and has significantly impacted their overall genome evolution and virulence (Feurtey *et al.* 2019). Stukenbrock argued for the need of additional experimental models to study hybridization, given its role in the exchange of virulence specificities and rapid emergence of new pathogenic species.

While human activity such as global trade, travel, and monoculture are driving the spread and emergence of fungal pathogens, environmental stresses are limiting crop yields and disturbing plant microbe symbioses. John Taylor (University of California, Berkeley, USA) found that the mutualistic association of sorghum, a major cereal crop and energy plant, and arbuscular mycorrhizal (AM) fungi is disrupted during drought (Varoquaux *et al.* 2019). Through a large-scale transcriptomic analysis of drought response in *Sorghum bicolor*, Taylor determined that the expression of *S. bicolor* genes critical for AM symbiosis are decreased and AM fungal mass is reduced during drought (Varoquaux *et al.* 2019). These results strongly indicate that drought diminishes the vital symbiotic relationship between plants and AM fungi, and highlight the potential of global climate change to perturb beneficial microbial associations.

A major obstacle in the study of AM fungi is the limited ability to culture these fungi axenically, thus impeding genomic and transcriptomic analyses. Jason Stajich (University of California, Riverside, USA) and colleagues at the University of Ottawa developed a method for large scale phylogenomics and transcriptomics using minute amounts of RNA from the spores of eight poorly studied, unculturable, AM fungi (Beaudet *et al.* 2018). Further, Stajich and collaborator Deborah Hogan (Geisel School of Medicine at Dartmouth, USA) investigated the mechanisms underpinning the evolution of drug resistance in an antifungal-naïve chronic *Candida lusitaniae* infection of a cystic fibrosis (CF) patient (Demers *et al.* 2018). Genome analysis of dozens of *C. lusitaniae* isolates from one CF patient revealed the highest number of nonsynonymous mutations in *MRR1* (Demers *et al.* 2018), which encodes a transcription factor capable of inducing fluconazole resistance in *Candida* species. The research team found that high Mrr1 activity conferred resistance to host and microbe factors, which suggested that drug resistance was selected for indirectly, and may explain the Mrr1 heterogeneity in this individual who had no prior azole exposure (Demers *et al.* 2018). Additionally, Stajich and members of Robert Cramer Jr’s research lab (Geisel School of Medicine at Dartmouth, USA) determined a link between filamentous biofilm morphology and virulence in the lung-infecting human fungal pathogen *Aspergillus fumigatus* (Kowalski *et al.* 2019). They found that under hypoxic conditions, *A. fumigatus* expressed a subtelomeric gene cluster that altered hyphal morphology and led to increased host inflammation, rapid disease progression, and mortality in a murine model of invasive aspergillosis (Kowalski *et al.* 2019).

## Identify mechanisms of fungal adaptation and interactions with hosts and other microbes

Fungi have a remarkable capacity to adapt to diverse environmental niches, ultimately allowing them to cause disease in an unparalleled range of evolutionarily distant hosts. While some fungi exhibit exquisite host specificity, others, such as *Fusarium* spp., *Aspergillus* spp., and *Cryptococcus* spp., possess the ability to infect animal, plant, and protozoan hosts (Fisher *et al.* 2012, 2016, 2018). Fungi can adapt to occupy new hosts via transfer from a similar or distantly related species or from the environment. While fungi originating from other hosts are adapted for host survival, fungi acquired from the environment have no prior requirement for the machinery to enable survival and replication within the host (Casadevall and Pirofski 2007). Arturo Casadevall (Johns Hopkins University, USA) proposed the concept of ‘dual use’ virulence factors to help explain the mechanism by which environmental fungi acquire and maintain virulence for animal hosts (Casadevall *et al.* 2003). ‘Dual use’ virulence factors are attributes that confer survival advantages in both animal hosts and the environment (Casadevall *et al.* 2003). This concept is exemplified by the soil fungus *Cryptococcus neoformans* which upon inhalation from the environment as spores can cause life-threatening infections in immunocompromised individuals. Casadevall found that the survival strategies of *C. neoformans* after ingestion by macrophages and amoebae were similar, and required the use of several ‘dual use’ virulence factors, such as expression of capsule and melanization (Steenbergen *et al.* 2001). These results suggest that *C. neoformans* mammalian virulence is a consequence of adaptations that evolved as protection against environmental predators such as amoebae (Steenbergen *et al.* 2001; Casadevall and Pirofski 2007).

Although our understanding of the mechanisms underlying fungal virulence in animal hosts is far from complete, it greatly outweighs our knowledge of fungal virulence strategies in plants. The corn smut fungus *Ustilago maydis* possesses more than 200 secreted virulence effector proteins with unknown molecular function (Lanver *et al.* 2017). These virulence effectors can either operate in the apoplast or be transferred to host cells via mechanisms that, until recently, remained elusive. Regine Kahmann (Max Planck Institute, Germany) has since discovered the *U. maydis* proteins that are likely responsible for virulence effector delivery to the host. By focusing on effectors that are expressed by the fungus during infection, Kahmann identified several fungal effectors and interacting fungal transmembrane proteins that are essential for virulence. She determined that these proteins assemble, and that assembly is necessary for suppressing pathogen-associated molecular pattern (PAMP) triggered host immunity. Further, she confirmed that the genes encoding these proteins are present in all smut fungi sequenced to date, implicating this assembly as a promising therapeutic target to combat smut fungi that infect economically important hosts such as corn, barley, wheat, oats, and sugarcane.

While fungi are the major pathogens for plants and non-mammalian animal species, a more limited number of fungi cause disease in mammals (Bergman and Casadevall 2010). Mammalian resistance to invasive fungal disease has been attributed to a combination of adaptive immunity and high body temperatures, which produce a thermal exclusionary zone for most fungal species (Bergman and Casadevall 2010). One concern with anthropogenic-caused global warming is that higher ambient temperatures will lead to the adaptation of fungal species to warmer temperatures and overcome the thermal barrier that protects mammals against many potential fungal pathogens (Garcia-Solache and Casadevall 2010). Using mathematical modeling, Sarah Gurr (University of Exeter, England) determined that plant fungal pathogen burden will increase, particularly in the northern hemispheres, with gradual climate warming (Bebber *et al.* 2013). Gurr investigated the impact of temperature warming on fungal pathogen adaptation experimentally and found that *Z. tritici* strains adapted to grow at elevated temperatures survived a sudden transition to temperatures exceeding that of the human body, which may reflect the potential of dramatic weather to acclimate pathogens to human body temperature.

While thermotolerance is a virulence trait shared among fungal pathogens of mammals, there is a vast diversity of factors that enable fungi to thrive and cause disease in the host. In the human fungal pathogen *C. neoformans*, iron acquisition is crucial to the deployment of major virulence factors, such as the polysaccharide capsule. James Kronstad (University of British Columbia, Canada) determined a role for the extracellular mannoprotein Cig1 in heme uptake, possibly through acting as a hemophore (Cadieux *et al.* 2013). He identified that loss of Cig1 in *C. neoformans* mutants lacking additional iron uptake systems attenuated virulence in a mouse inhalation model of cryptococcosis (Cadieux *et al.* 2013). Kronstad further explored the mechanisms of heme uptake by characterizing the role of clathrin-mediated endocytosis (Bairwa *et al.* 2019), and by making use of strains expressing a genetically encoded heme sensor. The latter approach identified additional contributions of endomembrane trafficking in the use of heme as a sole iron source and provided a tool to assess candidate antifungal drugs with heme-related activities. Parallel work on heme biosynthesis and uptake by *U. maydis* suggested that heme is not available to the fungus during proliferation in corn. In general, these studies provide evidence that inhibiting iron and heme acquisition may be a broadly effective antifungal strategy against both plant and animal fungal pathogens.

The ability to undergo morphological transitions is widespread in the fungal kingdom and often plays a crucial role in host invasion and pathogenesis. This is known to be the case for the human fungal pathogen *Candida albicans*, where strains locked as either yeast or hyphae are typically avirulent in a murine model of systemic infection (Lo *et al.* 1997; Saville *et al.* 2003). Leah Cowen (University of Toronto, Canada) explored the circuitry underlying temperature-dependent morphogenesis in *C. albicans* and identified the heat shock transcription factor Hsf1 and molecular chaperone Hsp90 as key regulators (Veri *et al.* 2018). Cowen determined that depletion of Hsf1 caused filamentation in the absence of elevated temperature by compromising Hsp90 function (Veri *et al.* 2018). Conversely, she found that overexpression of Hsf1 resulted in filamentation through an Hsp90-independent mechanism via expansion of Hsf1 direct targets that drives overexpression of positive regulators of filamentation (Veri *et al.* 2018). Cowen further interrogated the role of Hsp90 in *C. albicans* filamentation and found that genetic and pharmacological perturbation of Hsp90 resulted in increased protein levels of the 20S proteasome (O’Meara *et al.* 2019) and that compromising proteasome function induces *C. albicans* filamentation. In addition to temperature, morphogenesis in the host is influenced by factors such as host immune cells and the microbiota. Cowen investigated the genes required by *C. albicans* for morphogenesis in response to phagocytosis by macrophages and identified hundreds of genes necessary for this response. Further, she implicated a host-derived protein as a filamentation-inducing component within the macrophage. Cowen explored interkingdom interactions between *C. albicans* and *Lactobacillus* species and determined that *Lactobacilli* secrete a molecule that blocks *C. albicans* filamentation and immunopathology. These findings highlight the multitude of factors within the host that influence *C. albicans* morphogenesis and the complex genetic circuitry that underlies this important virulence trait.

## Understand the evolution of resistance to fungicides and antifungals across the fungal kingdom

There has been an unprecedented rise in the rate of emergence of pathogenic fungi resistant to the limited arsenal of fungicides and antifungals (Fisher *et al.* 2018). Notably, *Candida auris* is a new multidrug-resistant fungal pathogen first isolated in 2009, which exhibits limited susceptibility to antifungal agents. Global sampling and whole genome sequencing efforts have been undertaken by Christina Cuomo (Broad Institute, USA) in collaboration with the Centers for Disease Control and Prevention and others to study the genomic epidemiology of *C. auris*. Drug-resistance profiling of 304 *C. auris* isolates from 19 countries determined that 83% of isolates were resistant to one or more classes of antifungals, with resistance occurring most frequently to fluconazole. Genomic sequencing revealed that the majority of fluconazole-resistant isolates harbored mutations in the drug target *ERG11*, which in clades I and III often co-occurred with mutations in *TAC1* (Rybak *et al.* 2020). Further, tip-dating used to estimate ancestral divergences suggested that the date of emergence of drug-resistant subclades of *C. auris* occurred in the 1980s (Rybak *et al.* 2020), when azoles became widely used to treat fungal infections and were also made available for agricultural use (Sheehan *et al.* 1999; Morton and Staub 2008).

Drug resistance is widespread across the fungal kingdom and can arise via mechanisms such as alterations to the target, regulation of stress response pathways, upregulation of efflux, and genomic plasticity, which were highlighted by Neil Gow (University of Exeter, England) (Fisher *et al.* 2018). A novel form of RNA interference (RNAi)-based epigenetic drug resistance, termed epimutation, was recently discovered as a mechanism of FK506 resistance in the fungus *Mucor circinelloides* by Joseph Heitman (Duke University, USA) (Calo *et al.* 2014). He identified that RNAi is spontaneously triggered in *M. circinelloides* to silence expression of the drug target of FK506 and give rise to drug-resistant epimutants (Calo *et al.* 2014; Chang *et al.* 2019b). Heitman and colleagues showed that an alternative non-canonical RNA degradation pathway competes with epimutation, and that mutation of this alternative pathway enhances the frequency and stability of epimutations (Calo *et al.* 2017). Recently, they extended the impact of epimutation by showing that isolates resistant to 5-FOA can harbor epimutations in the *pyrF* or *pyrG* genes, which encode the enzymes that convert 5-FOA into its active toxic form (Chang *et al.* 2019a). Heitman determined that epimutants exhibited organ-specific stability in a murine model of *M. circinelloides* infection, with reversion to wild type occurring more rapidly in the brain compared to other organs, and increased epimutation in isolates recovered from infected animals, with implications for pathogenesis and drug resistance (Chang and Heitman 2019). These findings may provide insights into examples of unstable drug resistance that have been observed in other human fungal pathogens known to harbor active RNAi pathways (Stone *et al.* 2019).

## Develop novel strategies to thwart fungal disease

There is an urgent need for strategies to disarm fungi that threaten human health, biodiversity, and food security. Addressing this crisis will require the identification of drug targets, immunotherapies, biomarkers for risk stratification, disease intervention strategies, and ultimately, novel antifungals. In the quest for new therapies, Gerry Wright (McMaster University, Canada) developed a natural product library to identify compounds with antifungal, antibacterial, antiparasitic, and anticancer activities. He leveraged this library to discover Ibomycin, a complex macrolactone with antifungal activity against *C. neoformans* (Robbins *et al.* 2016) and butyrolactol A, which potentiates caspofungin against *C. neoformans*. Moreover, Wright established a pipeline for synthetic natural product synthesis to generate novel and diverse chemical entities through the expression of optimized biosynthetic genes in yeast. Wright has already demonstrated success in identifying compounds with antibacterial activity using this pipeline.

Identifying molecules with antifungal activity is only half the battle, as determining the precise cellular target of a compound can be equally challenging. Charles Boone (University of Toronto, Canada) developed a powerful chemical-genetic screening system in *Saccharomyces cerevisiae* to functionally annotate chemical libraries and map compounds to specific biological processes (Piotrowski *et al.* 2017). In this high-throughput system, a pool of barcoded deletion mutants for 310 genes covering all major bioprocesses are exposed to individual compounds and then sequenced to generate a chemical-genetic profile for each compound (Piotrowski *et al.* 2017). The use of a highly multiplexed barcode-sequencing protocol using 768 multiplex primers, each with a unique 10-bp tag, allows for the DNA barcodes from 768 different chemical-genetic experiments to be combined and sequenced simultaneously, enabling the assembly of thousands of chemical-genetic profiles (Piotrowski *et al.* 2017). Further, Boone advocated for the use of genome-wide mutant collections in *S. cerevisiae* to link bioactive compounds to cellular pathways or targets, which could readily be applied to determine the mode of action for compounds with antifungal activity.

Donald Sheppard (McGill University, Canada) has taken a more targeted approach to antifungal drug discovery, by exploiting fungal and bacterial glycoside hydrolases for the disruption of microbial biofilms (Snarr *et al.* 2017). Specifically, he determined that glycoside hydrolases Sph3 and PelA from *A. fumigatus* and *Pseudomonas aeruginosa*, respectively, disrupted *A. fumigatus* biofilms and reduced pulmonary fungal burden in a mouse model of invasive aspergillosis (Snarr *et al.* 2017). Further, Sheppard established a pipeline to develop anti-polysaccharide monoclonal antibodies for use as laboratory reagents, diagnostic tools, and potential therapies, such as CAR-T cell therapy for the treatment of *A. fumigatus* in post-transplant patients.

Fungal infections threaten global human health as single etiological agents of disease and as comorbidities with tuberculosis, AIDS, cancer, and respiratory illnesses. The widespread co-occurrence of serious fungal infections with these and other diseases highlights the dire need for biological and genetic markers to identify patients that would benefit from preventative therapeutic interventions. In recent years, there has been a growing appreciation of the burden of allergic bronchopulmonary aspergillosis (ABPA), a progressive fungal allergic lung disease that impacts over 5 million asthmatic people worldwide (Agarwal and Chakrabarti 2013), although risk-markers are lacking. David Denning (The University of Manchester, England) observed that patients with fungal asthma carrying a variant in the transcription factor *ZNF77* had higher *A. fumigatus* loads in their respiratory airways (Gago *et al.* 2018). Denning found that human epithelial cells engineered to harbor this genetic variant lost epithelial monolayer integrity and had increased levels of extracellular matrix proteins, which promoted *A. fumigatus* conidial adhesion, germination, and growth (Gago *et al.* 2018). Overall, these changes made cells carrying the *ZNF77* variant more receptive to *A. fumigatus* infection, suggesting that *ZNF77*-genotyping of patients with asthma may be useful as a risk-marker for ABPA (Gago *et al.* 2018).

In addition to their devastating impact on human health, fungi are causing mass mortalities in wildlife and driving rapid loss of biodiversity. Akin to the decimation of amphibian populations by *B. dendrobatidis*, there have been massive die offs in North American hibernating bat populations due to white-nose syndrome (WNS) caused by the introduction of a single clone of the fungus *Pseudogymnoascus destructans* (Trivedi *et al.* 2017). Since its recent emergence in New York State in approximately 2006 (Blehert *et al.* 2009), there has been substantial progress in understanding the etiology of WNS and developing vaccines with efficacy against *P. destructans* (Rocke *et al.* 2019). David Blehert (U.S. Geological Survey National Wildlife Health Center) described the histopathologic criteria to confirm WNS in bats (Meteyer *et al.* 2009) and proposed a multi-stage disease progression for WNS, linking wing tissue damage by *P. destructans* to morbidity and mortality (Warnecke *et al.* 2013; Verant *et al.* 2014). In this model, wing damage in hibernating bats leads to more frequent arousals from torpor, resulting in loss of blood electrolyte and respiratory homeostasis, dehydration, and depletion of fat reserves, which ultimately causes mortality when energy reserves become exhausted (Warnecke *et al.* 2013; Verant *et al.* 2014).

To enable further molecular characterization of bat WNS, Bruce Klein and his graduate student Marcos Isidoro-Ayza (University of Wisconsin-Madison, USA) generated a keratinocyte cell line (MyluK) from the little brown bat (*Myotis lucifugus*) as an *in vitro* skin model to study *P. destructans* infection. He identified that MyluK cells exhibit defects in the innate immune response to *P. destructans* compared to infection by *Athroderma redellii*, an endemic fungus that causes a non-lethal infection in hibernating bats (Lorch *et al.* 2015). Klein found that MyluK dectin-1 recognized *A. redellii* spores more strongly than *P. destructans*, indicating differences in the keratinocyte immune response to these two fungi. As a further effort to develop therapeutic interventions for bat WNS, Klein and others at the University of Wisconsin-Madison and the U.S. Geological Survey National Wildlife Health Center generated a vaccine with efficacy against *P. destructans* (Rocke *et al.* 2019). Specifically, they found success with a vaccine that uses an attenuated raccoon poxvirus as a vector to express *P. destructans* calnexin and serine protease destructin-1 as immunogenic antigens (Rocke *et al.* 2019). Bats treated with this vaccine developed WNS at a lower rate (Rocke *et al.* 2019), and work is ongoing to develop strategies for vaccine distribution.

## Conclusions and outlook

Although substantial progress has been made toward understanding the forces driving the emergence of fungal pathogens, the mechanisms underlying fungal adaptation, and the evolution of drug resistance, much remains to be done. We have yet to leverage fundamental discoveries to develop a new class of antifungal in over 20 years or establish a resistance-evasive strategy to protect crops from devastating fungal pathogens. As highlighted here and in the review *Threats posed by the Fungal Kingdom to humans*, *wildlife*, *and agriculture* (Fisher *et al.*, 2020 in press), there is a dire need for coordinated international research to address these challenges and mitigate the devastating impact of fungal pathogens on ecosystem and human health. By uniting experts on plant and animal fungal pathogens in a sustained manner, the CIFAR *Fungal Kingdom* program is poised to accelerate the pace of discovery and develop innovative approaches to thwart fungal threats.
